# Effect of ice water injection toward the duodenal papilla for preventing post-ERCP pancreatitis: study protocol for a multicenter, single-blinded, randomized controlled trial (EUTOPIA study)

**DOI:** 10.1186/s12876-022-02462-z

**Published:** 2022-08-12

**Authors:** Shunjiro Azuma, Akira Kurita, Kenichi Yoshimura, Tomoaki Matsumori, Yosuke Kobayashi, Kei Yane, Osamu Inatomi, Kenji Sawada, Ryo Harada, Shujiro Yazumi

**Affiliations:** 1 Department of Gastroenterology and Hepatology, Kitano Hospital, Tazuke Kofukai Medical Research Institute, 2-4-20 Ohgimachi, Kita-ku, Osaka, 530-8480 Japan; 2 Department of Gastroenterology and Hepatology, Rakuwakai Otowa Hospital, 2 Otowachinjicho Yamashina-ku, Kyoto, 607-8062 Japan; 3 Medical Center for Translational and Clinical Research, Hiroshima University Hospital, 1-2-3 Kasumi Minami-ku, Hiroshima, 734-8551 Japan; 4 Department of Gastroenterology and Hepatology, Kyoto University Hospital, 54 Shogoin Kawaharacho, Sakyo-ku, Kyoto, 606-8507 Japan; 5 Department of Gastroenterology, Seirei Hamamatsu General Hospital, 2-12-12 Sumiyoshi, Naka-ku, Hamamatsu-shi, Shizuoka, 430-8558 Japan; 6 Department of Gastroenterology and Hepatology, Tonan Hospital, 3-8, 7-3, Kita 4-jo Nishi, Chuo-ku, Sapporo-shi, Hokkaido, 060-0004 Japan; 7 Department of Gastroenterology, Shiga University of Medical Science, Seta Tsukinowa-Cho, Otsu city, Shiga 520-2192 Japan; 8 Department of Gastroenterology and Hepatology, Japanese Red Cross Osaka Hospital, 5-30 Fudegasaki-cho, Tennoji-ku, Osaka, 543-8555 Japan; 9 Department of Gastroenterology and Hepatology, Japanese Red Cross Okayama Hospital, 2-1-1 Aoe, Kita-ku, Okayama City, Okayama 700-8607 Japan

**Keywords:** ERCP, PEP, Post-ERCP pancreatitis, Ice water

## Abstract

**Background:**

Endoscopic retrograde cholangiopancreatography (ERCP) is an essential procedure in the diagnosis and treatment of biliopancreatic diseases. The most common adverse event of ERCP is post-ERCP pancreatitis (PEP), which can sometimes be severe. Our previous study suggested that injecting ice water at the end of ERCP suppressed PEP, and we decided to investigate this effect in a multicenter randomized controlled trial.

**Methods:**

This study is being conducted at eight hospitals in Japan starting in April 2022. Patients undergoing ERCP will be randomized to ice water group and control group. In the ice water group, 250 ml of ice water is injected toward the papilla at the end of ERCP. The next morning, a physical examination and blood tests are performed to evaluate for the development of pancreatitis. The goal is to have 440 cases in each group.

**Discussion:**

The main cause of PEP is thought to be papilla edema. Cooling the papilla, as everyone naturally does at the time of a burn, is expected to prevent its inflammation and edema. Various methods to suppress PEP have been reported, but so far none of them are reliable. The method we have devised is very simple, easy, and safe. We hope that our study will change the world's ERCP common practice.

*Trial registration*:UMIN000047528. Registered 20 April 2022, https://center6.umin.ac.jp/cgi-open-bin/ctr_e/ctr_view.cgi?recptno=R000053209

## Background

Endoscopic retrograde cholangiopancreatography (ERCP) is indispensable for the diagnosis and treatment of biliopancreatic diseases; post-endoscopic retrograde cholangiopancreatography pancreatitis (PEP) is the most problematic procedure-related adverse event. The incidence of PEP is approximately 3.5–9.7%, with severity and mortality rates of 0.04–0.2% and 0.1–0.7%, respectively [1]. Risk factors for PEP include sphincter of Oddi dysfunction (SOD), female sex, history of pancreatitis, history of PEP, difficulty in bile duct cannulation, insertion of a guidewire into the pancreatic duct, and contrast injection into the pancreatic duct [1]. The main cause of PEP is thought to be papillary edema associated with the procedure. Endoscopic techniques, such as prophylactic pancreatic stenting [2–4], wire-guided cannulation [5, 6], and the use of rotatable catheters [7], have been reported to be effective methods for preventing PEP; however, they are not reliable. Various drugs have been studied to suppress papillary edema, but currently, none has been shown to be useful except nonsteroidal anti-inflammatory drugs (NSAIDs) suppositories [8–10]. Prophylactic administration of NSAIDs suppositories into the rectum prior to ERCP has been reported to significantly reduce the incidence of PEP [11–13] and is now widely used. However, NSAIDs are contraindicated in patients with renal failure, aspirin asthma, gastric ulcers, in the elderly, or in patients with allergies.

For prevention of PEP, we are investigating a new and simple method that can be performed by anyone. Cooling is widely known to be effective in treating acute inflammation and edema in burns. Similarly, we hypothesized that cooling the papilla of Vater would help reduce papillary edema. Our previous single-center prospective study suggested that cooling the papilla with ice water may reduce the incidence of PEP by 4% [14]. However, the results of this trial are uncertain because it is only a single-arm prospective study. This uncertainty has led to a call for randomized controlled trials to validate the results.

### Objectives

#### Primary objective

To determine the effect of ice water injection into the papilla of Vater on the incidence of PEP.

#### Secondary objective

This study aims to determine the efficacy of ice water injection into the papilla of Vater on the incidence of moderate-to-severe PEP and other adverse events.

## Methods/design

### Trial design

EUTOPIA is a multicenter, randomized, controlled, patient-blinded, superiority trial with two parallel groups.

### Participants

#### Study settings

The EUTOPIA study is being conducted in eight hospitals (two universities and six general hospitals) in Japan.

#### Eligibility criteria

**Fig. 1:**
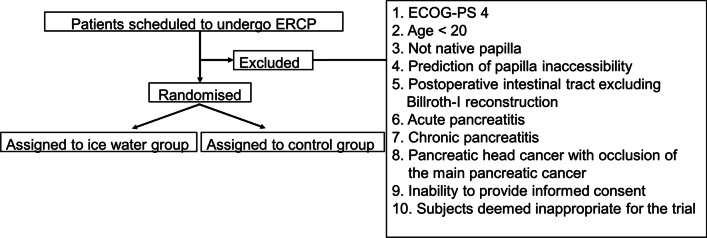
Eligibility criteria and grouping

**Fig. 2:**
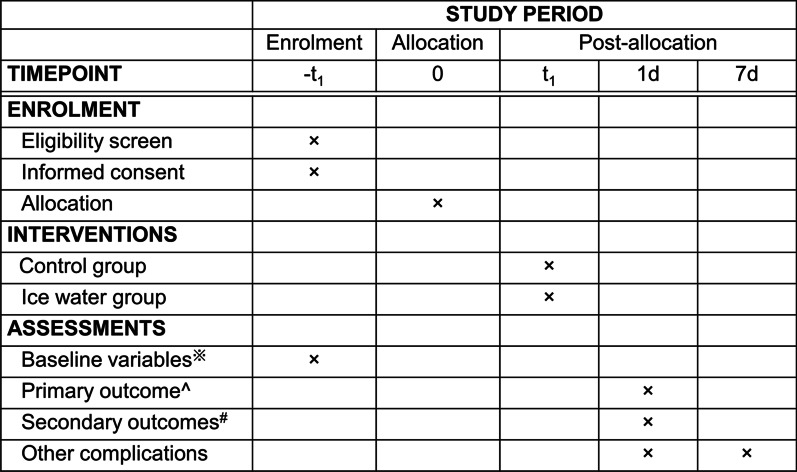
Study protocol. ^※^Baseline variables: age, sex, ECOG-PS, ASA-PS, history of acute or recurrent pancreatitis, serum total bilirubin level before ERCP, serum amylase level before ERCP, ERCP indication, and presence of SOD, cholangitis, pancreatic duct obstruction at the head of the pancreas. ^Primary outcome: presence of PEP. ^#^Secondary outcomes: presence of PEP in cases of difficult cannulation, PEP by various ERCP procedures, PEP by the presence of pancreatic duct cannulation and pancreatography, PEP by cannulation time, moderate and severe PEP, and PEP by high-risk factors for PEP

Figure 1 shows the patients’ eligibility criteria and Fig. 2 shows the study protocol.

#### Inclusion criteria

Patients over 20 years of age with native papilla who undergo ERCP are eligible.

#### Exclusion criteria

Patients fulfilling one or more of the following criteria are excluded:

(1) Eastern Cooperative Oncology Group Performance Status 4; (2) age younger than 20 years; (3) non-native papilla; (4) prediction of papilla inaccessibility; (5) postoperative reconstructed intestinal tract excluding Billroth I reconstruction; (6) presence of acute pancreatitis; (7) presence of chronic pancreatitis; (8) presence of pancreatic head cancer with occlusion of the main pancreatic duct; (9) inability to provide written informed consent; and (10) patients deemed inappropriate for the trial.

### Interventions

A diagram of the study protocol is shown in Fig. 2. All patients are fasted on the day of ERCP. NSAIDs suppositories are not administered. During the examination, the heart rate, non-invasive blood pressure, and oxygen saturation are monitored, and pain is reduced using sedatives and analgesics.

The patients are randomized into the ice water or control group prior to ERCP.

ERCP is performed using a side-viewing duodenoscope (TJF-260 V or TJF-Q290V; Olympus Medical Systems Co. Ltd., Tokyo, Japan) in a standard manner.

In the ice water group, a total of 250 mL of ice water is injected, which is done in five increments using a 50 mL syringe, toward the papilla at the end of ERCP. Duodenal fluid is aspirated after each 50-mL injection of ice water, and the injection is repeated.

**Fig. 3:**
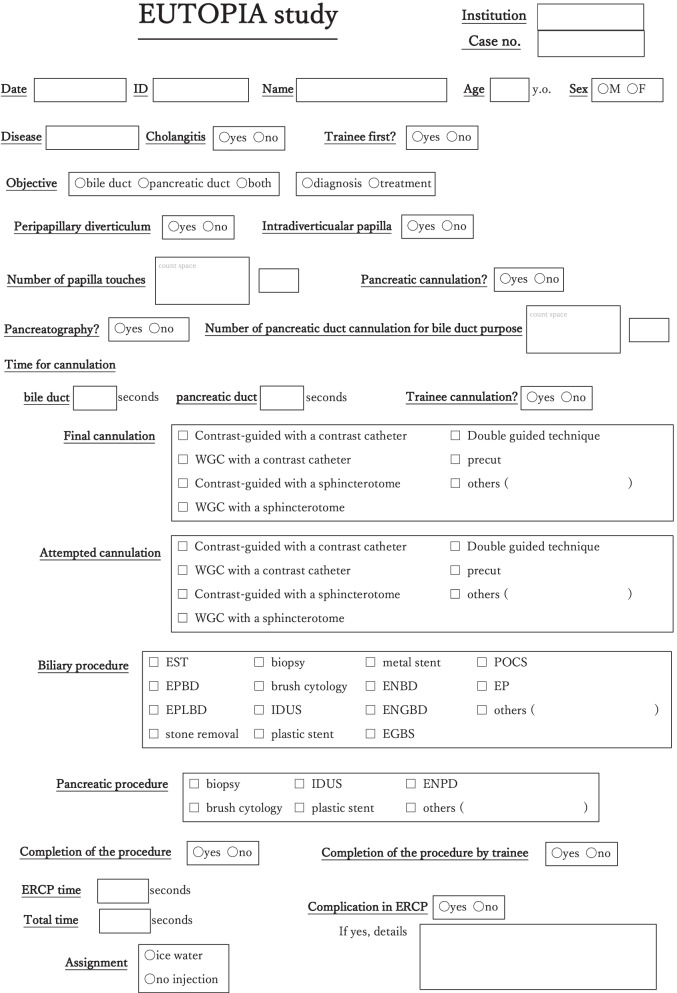
Findings form

During ERCP, the total examination time (from insertion of the scope to its removal), procedure time (from the start of cannulation to the end of treatment), time required for cannulation, and the number of papillary contacts, etc. are recorded on the findings form (Fig. 3).

The patient’s symptoms are monitored after ERCP. The next morning, medical examination is performed to determine if the patient meets the criteria for PEP, and if the patient experiences abdominal pain, blood tests may be performed at the discretion of the physician in charge.

### Outcomes

#### Primary outcome measure

The primary outcome is the incidence of PEP in the ice water and control groups. PEP is defined as the onset of abdominal pain within 24 h of ERCP and elevation of serum amylase and lipase levels to at least three times the upper limit of normal based on the Cotton criteria[15].

#### Secondary outcome measures

The secondary outcomes are as follows: (1) incidence of PEP in cases of difficult cannulation, (2) incidence of PEP by various ERCP procedures, (3) incidence of PEP by the presence of pancreatic duct cannulation and pancreatography, (4) incidence of PEP by cannulation time, (5) incidence of moderate and severe PEP, (6) incidence of PEP by high-risk factors for PEP, and (7) incidence of other complications.

### Definitions

The severity of PEP is classified as follows, modifying Cotton criteria: mild disease, which requires 2–3 days of fasting; moderate disease, which requires 4–10 days of fasting; and severe disease, which requires 11 or more days of fasting[15]. In addition to the above, patients with necrosis or pseudocyst formation or those who underwent percutaneous drainage or surgery were defined to have severe disease[15].

Difficult cannulation is defined as contact with the papilla more than five times, cannulation time more than 5 min, and unintentional pancreatic duct cannulation more than two times [1]. Definition and severity of cholangitis prior to ERCP; in accordance with Tokyo Guidelines 2018[16]. ERCP trainee is defined as a physician with less than 200 cases of ERCP experience. The following factors have been identified as risk factors for PEP: (1) pre-cut sphincterotomy, (2) endoscopic pancreatic sphincterotomy, (3) endoscopic papillary balloon dilation, (4) difficult cannulation cases, (5) pancreatography, (6) female patients under 60 years old, (7) suspected SOD, (8) history of recurrent pancreatitis, and (9) history of PEP[1].

Regarding other ERCP comorbidities, hemorrhage is defined as hematemesis or a drop in hemoglobin concentration of > 2 g/dL and perforation as the presence of air or intestinal contents beyond the intestinal tract[17]. Other comorbidities and their severity follow the ASGE guidelines[17].

The ice water used in this study is defined as 250 mL of chilled water in a refrigerator with ten ice cubes made with an ice machine.

### Sample size

#### Number of patients

Without any form of prophylaxis, the incidence of PEP in the native papilla can reach 10–15% as reported in previous studies[1, 11, 18, 19]. Our previous study revealed that ice water injection reduced the incidence of PEP from 11 to 4%, and the relative risk reduction was 63.6%[14]. Assuming a baseline PEP risk of 10% in normal native papilla, a two-sided α = 0.05 and a power of 0.8, 435 patients per study arm are required to detect a 50% reduction in the incidence of PEP to 5%. This absolute reduction in incidence is believed to be clinically relevant and substantial enough to change the existing clinical practice. We aim to enroll 440 patients into each group to accommodate patients who will be lost to follow-up, missing data, or withdrawal of consent.

### Recruitment

Patient inclusion started in May 2022 in eight Japanese hospitals. Enrollment in ongoing.

### Assigning interventions

#### Allocation

Randomization is centralized, web-based, and accessible 24 h a day; it is balanced (1:1) and stratified by center and the cause of the indication for ERCP [for bile duct cannulation or otherwise (for pancreatic duct and both bile and pancreatic ducts cannulation)].

#### Sequence generation

The randomization sequence is generated by a professional technician from Hyogo University who is not involved in patient recruitment. The sequences are implemented using the software used for data collection.

#### Blinding

The allocation result is unknown to the patient because knowing it may affect the appearance of abdominal pain, which predicts the onset of PEP.

### Data collection, management, and analysis

#### Data collection and management

The study data are recorded in an electronic web-based case report form (eCRF) from the medical records of each patient (source data) by the trial site personnel. The data manager, in cooperation with the coordinating investigator, established the trial database by exporting data from the eCRF. Any protocol deviations are recorded in either the eCRF or the medical records.

#### Statistical analysis

The full analysis set (FAS) is the population of patients enrolled in the study, excluding duplicate or erroneous enrollments, cases of inadequate study treatment, and cases in which no post-assignment data are available.

The per-protocol set (PPS) excludes FAS cases in which efficacy could not be assessed due to inadequate observation, etc., and cases of serious deviation from or violation of the study protocol.

For each allocation group, the percentage of PEP occurrences will be calculated using the number of FAS cases as the denominator. The exact 95% confidence intervals (CI) of Clopper and Pearson will be calculated. The frequencies, expression proportions, and 95% CI will also be calculated for each group. Fisher's exact test will be used to compare the groups. The test will be two-tailed with a significance level of 5%. The same analysis will be performed for PPS as a reference.

After dividing the patients into subgroups according to background factors, comparisons between the groups will be made. Binary data (PEP and other comorbidities) will be evaluated using odds ratios and 95% CI, and compared using Fisher's exact test. When the background factor is a continuous variable, subgroups will be created based on the median value. The results of the subgroup analysis will be graphically represented by a forest plot.

### Data monitoring

The research is monitored to ensure that it is properly conducted for credibility and to protect the participants. Monitoring procedures shall be prepared, and one person shall be designated to monitor the progress of the relevant clinical research and whether or not, it is being conducted in accordance with the implementation plan and research protocol.

The monitoring supervisor shall conduct monitoring, paying attention to (a) through (d).

- The human rights of research participants are protected and their safety is ensured.
- The clinical research is being conducted in compliance with the latest implementation plan and research protocol.
- Consent to conduct the clinical research is obtained in writing from the research participants.
- The accuracy of records is verified in light of the original data.

If deemed necessary based on the results of monitoring or information from the research office, consideration will be given to confirming the results of the monitoring by means of telephone calls or visit to each participating facility and to providing information to other principal investigators. The principal investigators will endeavor to resolve problems as early as possible by providing feedback to research supervisors.

## Discussion

We previously investigated the safety and efficacy of injecting iced water into the duodenum at the end of ERCP to decrease PEP [14]. As a result, we verified its safety. As for efficacy, we could not show a significant difference, although there was an increased tendency to decrease PEP in the iced water group (4%) than in the control group (11%). However, the previous study had the limitation of not being a direct comparative study and having a small number of participants. Therefore, we are conducting a direct comparative study in a multi-center setting in to prove its usefulness and to provide a high level of evidence.

The primary cause of PEP may be papillary edema. By cooling the papilla, as usually done in cases of burns, we hope to prevent inflammation and edema. The mechanism by which cooling prevents edema is not well understood, but it is speculated that cooling reduces the amount of thermal energy imparted on the tissue, thereby reducing damage [20].

Prophylactic pancreatic stenting, wire-guided cannulation, and rotatable catheters have been reported as methods for reducing PEP. In addition, the prophylactic administration of NSAIDs suppositories into the rectum prior to ERCP to suppress PEP is becoming more common. However, all these methods are labor-intensive, costly, and have the potential for adverse events associated with them. We are investigating a method that is safe, simple, and convenient.

This study has some limitations. We are unable to finely control the duodenal temperature because it is impossible to monitor the temperature at all times, especially when performing ERCP at our institution. This makes it impossible to assess the relationship between the temperature of the injected water and the temperature in the duodenum, although it has been reported that cooling at a very low temperature in burns is counterproductive [21].

Our goal is to prove that papilla cooling at the end of ERCP reduces PEP. We believe that papilla cooling can reduce the worldwide incidence of PEP if our hypothesis is proven.

## Data Availability

The datasets used and/or analyzed during the current study are available from the corresponding author on reasonable request.

## Abbreviations

ERCP: Endoscopic retrograde cholangiopancreatography
PEP: Post-endoscopic retrograde cholangiopancreatography pancreatitis
SOD: Sphincter of Oddi dysfunction
NSAIDs: Nonsteroidal anti-inflammatory drugs
CT: Computed tomography
eCRF: Electronic web-based case report form
FAS: Full analysis set
PPS: Per-protocol set
CI: Confidence interval
